# Prognostic efficacy of the human B-cell lymphoma prognostic genes in predicting disease-free survival (DFS) in the canine counterpart

**DOI:** 10.1186/s12917-016-0919-x

**Published:** 2017-01-09

**Authors:** Mohamad Zamani-Ahmadmahmudi, Sina Aghasharif, Keyhan Ilbeigi

**Affiliations:** 1 Department of Clinical Science, Faculty of Veterinary Medicine, Shahid Bahonar University of Kerman, P.O Box: 76169133, Kerman, Iran; 2 Department of Clinical Science, Faculty of Veterinary Medicine, Islamic Azad University, Garmsar Branch, Garmsar, Iran

**Keywords:** Canine B-cell lymphoma, Prognosis, Cox proportional-hazard analysis, Survival

## Abstract

**Background:**

Canine B-cell lymphoma is deemed an ideal model of human non-Hodgkin’s lymphoma where the lymphomas of both species share similar clinical features and biological behaviors. However there are some differences between tumor features in both species. In the current study, we sought to evaluate the prognostic efficacy of human B-cell lymphoma prognostic gene signatures in canine B-cell lymphoma.

**Methods:**

The corresponding probe sets of 36 human B-cell lymphoma prognostic genes were retrieved from 2 canine B-cell lymphoma microarray datasets (GSE43664 and GSE39365) (76 samples), and prognostic probe sets were thereafter detected using the univariate and multivariate Cox proportional-hazard model and the Kaplan–Meier analysis. The two datasets were employed both as training sets and as external validation sets for each other. Results were confirmed using quantitative real-time PCR (qRT-PCR) analysis.

**Results:**

In the univariate analysis, *CCND1, CCND2, PAX5, CR2, LMO2, HLA-DQA1, P53, CD38, MYC-N, MYBL1*, and *BIRCS5* were associated with longer disease-free survival (DFS), while *CD44, PLAU*, and *FN1* were allied to shorter DFS. However, the multivariate Cox proportional-hazard analysis confirmed *CCND1* and *BIRCS5* as prognostic genes for canine B-cell lymphoma. qRT-PCR used for verification of results indicated that expression level of *CCND1* was significantly higher in B-cell lymphoma patients with the long DFS than ones with the short DFS, while expression level of *BIRCS5* wasn’t significantly different between two groups.

**Conclusion:**

Our results confirmed *CCND1* as important gene that can be used as a potential predictor in this tumor type.

**Electronic supplementary material:**

The online version of this article (doi:10.1186/s12917-016-0919-x) contains supplementary material, which is available to authorized users.

## Background

Lymphoma is one of the most common malignancies in dogs and occurs in different forms, including multicentric, mediastinal (thymic), alimentary, cutaneous, and solitary types [1, 2]. Investigators have proposed canine B-cell lymphoma as a suitable model of human non-Hodgkin lymphoma (NHL) because the tumors of both species have common clinical manifestations and biological properties. However there are some differences between tumor features in both species [3, 4].

Some clinical and histological features have been proposed as prognostic factors in canine lymphoma [2, 5, 6]. For example, there are conflicting data on the use of the Kiel and Working Formulation classifications insofar as studies have revealed that both classifications are unreliable prognosticators [2, 5]. Nonetheless, in a study by Teske et al. (1994), the Working Formulation classification and Kiel classification were suggested as prognostic factors for the overall survival and time-to-relapse in treated dogs with malignant lymphoma, respectively [7]. Moreover, investigations have reported that such clinical parameters as age, sex, animal weight, and clinical stage have no robust efficiency for predicting overall survival and disease-free survival (DFS) times [5]. Some cellular proliferation markers such as Ki-67, PCNA, and AgNOR have been evaluated as suitable prognosis predictor. Indeed, Ki-67 and AgNOR have been reported as appropriate prognostic markers in human and canine malignant lymphoma [5, 8, 9], where AgNOR can be utilized for the grading of the canine and human NHL [10, 11].

Molecular phenotyping is a robust method for the definition of tumor subtypes and the detection of prognostic gene genes [12–15]. For instance, the gene expression profile analysis divided human diffuse large-B-cell lymphoma (DLBCL) into 3 distinct subtypes: activated germinal center-like B-cell lymphoma, B-cell lymphoma, and peripheral mediastinal B-cell lymphoma [12]. A similar investigation classified canine malignant lymphoma based on molecular profiling [6]. In different studies, 36 genes have been suggested as prognostic markers for human B-cell lymphoma (majorly DLBCL) (Table 1). To the best of our knowledge, there is limited information on the prognostic efficacy of these important gene markers in canine B-cell lymphoma as an ideal model of human NHL. In the present study, the robustness of these genes for the prediction of DFS in 2 canine B-cell lymphoma microarray datasets was investigated using the univariate/multivariate Cox proportional-hazard model and the Kaplan–Meier analysis. The prognostic efficacy of selected gene(s) in each dataset was validated via the other dataset.

**Table 1:** List of human B-cell lymphoma prognostic genes used in our study

BCL2 [1–4]	Ki-67 [5]
BCL6 [4, 6, 7]	LMO2 [4, 8]
BCL7A [4]	LRMP [4]
BIRC5 [9]	MYBL1 [4]
CCND1 [10]	MYCN [6]
CCND2 [8, 11]	NPM3 [6]
CD10 [4]	NR4A3 [12]
CD38 [4]	P53 [13]
CD44 [14]	PAX5 [15]
CFLAR [4]	PDE4B [12]
CR2 [4]	PIK3CG [4]
EEF1A1L4 [6]	PLAU [6]
FN1 [6]	PMS1 [4, 11]
HGAL [4, 6]	PRDM1 [11]
HLA-DQA1 [6]	SCYA3 [8, 11]
HLA-DRA [6]	SLA [4]
ICAM1 (CD54) [16]	SLAM [4]
IRF4 [4]	WASPIP [4]

References were provided in Additional file 1

## Methods

### Microarray expression datasets

Two canine B-cell lymphoma microarray datasets, namely GSE43664 [16] and GSE39365 [6] (platform: GPL3738), were obtained from the GEO database (http://www.ncbi.nlm.nih.gov/geo/). Expression data were downloaded in the CEL file format. The GSE43664 and GSE39365 datasets comprised 58 and 36 samples, respectively, where the GSE43664 samples were solely canine B-cell lymphoma (mainly diffuse large B-cell lymphoma [DLBCL]) and the GSE39365 samples contained both B-cell (*n* = 18) and T-cell lymphoma (*n* = 18). In the GSE39365 dataset, only B-cell lymphoma samples were included in the study. B-cell lymphoma samples in GSE43664 included DLBCL (mainly), MZL, and unknown. Additionally, B-cell lymphoma samples in GSE39365 included DLBCL (mainly), MZL, and BL. The clinical features of the studied cases are summarized in Additional file 1: Table S1 and S2. The data were first converted into expression values and then transformed logarithmically using the Affy package [17] in R environment, version 3.0.2 (http://www.r-project.org/). Survival time, compared using Student's t-test between two datasets (GSE43664: 9.6 ± 8.7 months and GSE39365:11.7 ± 12.1 months), wasn’t statistically different (*P* = 0.42).

### Extraction of prognostic gene expression values

Thirty-six human-specific genes, presumed as prognostic genes, were tested in the current study (Table 1). The literatures were mined to retrieve papers exploring prognostic genes or gene signatures in human B-cell lymphoma. Public databases (especially PubMed) were screened for papers describing genes predicting survival in human B-cell lymphoma. Finally, 36 genes were extracted from papers, where some of these genes weren’t evaluated as a single prognostic gene and proposed as a prognostic gene signature with the other genes. So, to perform a comprehensive assessment, we included all genes in our analysis. The corresponding probe sets of these genes and the related expression value for each probe set were retrieved from both datasets using MATLAB 7.8.0 (R2009a) (MathWorks, Natick, MA).

### Survival analysis and external validation

Survival analysis was performed using *Survival* (http://cran.r-project.org/package=survival) and *Survcomp* [18] packages in R environment. The Cox proportional-hazards analysis was used for constructing a model for the prediction of survival. In this analysis, the association between a group of covariates (genes) and the response variable (DFS) was evaluated. Two datasets were employed as training and validation (test) groups, where important prognostic gene(s) was identified in a group (training group) and then validated in the other dataset (validation group). We used an external validation instead of internal validation, as the former is generally more robust to the overfitting problem [19].

First, the univariate Cox analysis was performed and genes with a z score greater than 1.5 or less than -1.5 [13, 20] were selected for the multivariate Cox analysis, where a negative score and a positive score associated with longer and shorter survival respectivley. In the multivariate Cox analysis, statistically significant genes were entered into the analysis and significant covariate(s) was detected at a *P*-value lower than 0.05. Survival curves were depicted by Kaplan–Meier method and compared using the log-rank test. Furthermore, some clinical prognosis parameters such as animal age, sex, and tumor grade (high or low) (Additional file 1: Table S2) were assessed in the Cox analysis to determine their roles in the prediction model.

Next, the external validation of the resulted prognostic genes was determined. The prognostic gene(s) in each group was tested in the other group via the Kaplan-Meier method and the log-rank test. In addition, the expression of the prognostic genes were compared in human ABC-like (activated B-cell like) and human GCB-like (germinal center B like) groups, because GCB-like and ABC-like cases are associated with better and poorer prognoses, correspondingly [21]. For this analysis, the patients were categorized as GCB-like and ABC-like groups based on 1,180 canine-specific differentially expressed probe sets proposed by Richards et al. (2013) [16]. Grouping was carried out using the hierarchal clustering analysis provided in geWorkbench 2.5.1 package [22]. Subsequently, the expressions of the prognostic genes were compared between the two groups using the Student's t-test analysis provided in geWorkbench 2.5.1 package.

### Verification of the results by quantitative real-time PCR (qRT-PCR)

qRT-PCR procedure was performed as previously described [23, 24] on lymph node biopsy samples obtained from 60 dogs with B-cell lymphoma. All applicable international, national, and/or institutional guidelines for the care and use of animals were followed. Biopsy samples were processed using hematoxylin and eosin (H&E) staining method for the routine histopathology evaluation. Samples were diagnosed and subtyped based on the World Health Organization classification of hematopoietic and lymphoid tissues [25]. CD79a and CD3 antibodies (Dako, Denmark) were used for the confirmation of B-cell phenotype. CD79a-positive and CD3-negative samples were selected for subsequent analysis. Because mean survival time of GEO datasets samples that had lower expression (short survival) and higher expression (long survival) values than *CCND1* or *BIRCS5* median value were 6.9 months and 12.1 months respectively (see results), the selected cases for qRT-PCR included 30 dogs with DFS <7 months and 30 dogs with DFS >12 months. Mean age of the dogs with DFS >12 months and dogs with DFS <7 months were 8.3 years (range: 3-12 years) and 7 years (range: 2-10 years) respectively.

In brief, total RNA was extracted using Tripure isolation reagent (Roche, Germany) according to the manufacturer's protocol. cDNA was synthesized using Maxime RT PreMix Kit (Intron biotechnology, Korea) according to the manufacturer's instructions. The cDNA synthesis reaction was run at 45 °C for 60 min, followed by 95 °C for 5 min. Synthesized cDNA was used for final PCR assay. SYBR green-based quantitative real-time PCR (qRT-PCR) was performed using the Applied Biosystems 7500 Real- Time PCR system. Cycle conditions were 95 °C for 10 minutes, followed by 40 cycles of 95 °C for 15 s, 52 °C for 45 s, and 72 °C for 1 min. Data were analyzed by SDS 2.0 software (Applied Biosystems). Specific primers used for *CCND1* and *BIRCS5* were presented in Additional file 1: Table S3. HPRT was used as the reference gene for normalization of target gene expression. Comparative ΔCT-method was used for calculation of relative expression of the target gene [23]. Data are presented as fold change in gene expression level of the target gene. Fold changes in gene expression was compared between two groups (DFS <7 months vs. DFS >12 months) by Student's t-test. A *P* value lower than 0.05 was considered significant.

## Results

Probe sets corresponding to the prognostic genes were obtained from both datasets and subjected to subsequent survival analysis. Ninety one probe sets corresponding to 36 genes were retrieved from the each datasets. In the univariate analysis, the genes with a z score higher than 1.5 or lower than -1.5 were selected for the multivariate analysis. The results of the univariate analysis are summarized in Table 2. In the 58-sample dataset, *CCND1, CCND2, PAX5, CR2, BCL2L14, LMO2, HLA-DQA1, P53, MYC-N*, and *BIRCS5* had z scores lower than -1.5, which is associated with longer DFS. Conversely, *CD44, PLAU,* and *FN1* had positive z scores (higher than 1.5), which is correlated with shorter DFS. Moreover, in the 18-sample dataset, *CCND1, BIRCS5, MYC-N, LMO2, MYBL1*, and *CD38* had significant negative z scores (lower than -1.5). No genes with a z score higher than 1.5 was detected in the univariate analysis of the GSE39365 dataset (Table 2). Our subsequent multivariate analysis indicated that *CCND1* was a robust predictor in both datasets. Furthermore, *BIRCS5* in the GSE39365 dataset reached a statistically significant level (Table 3).

**Table 2:** Univariate Cox proportional-hazard analysis of B-cell lymphoma prognostic gene signatures in GSE43664 and GSE39365 datasets

	Coef	Exp (coef)	SE (coef)	z score	*P*
*GSE43664 dataset*					
*Cfa.21188.1.S1_s_at: (CCND2)*	-0.672	0.511	0.207	-3.24	0.0012
*Cfa.19972.1.S1_at: (BCL2L14)*	-3.44	0.0321	1.26	-2.72	0.0065
*CfaAffx.18137.1.S1_at: (CR2)*	-0.902	0.406	0.371	-2.43	0.015
*CfaAffx.4397.1.S1_x_at: (PAX5)*	-1.03	0.357	0.446	-2.31	0.021
*Cfa.37.1.S1_at: (BIRC5)*	-1.44	0.236	0.627	-2.3	0.021
*Cfa.16248.1.S1_at: (CCND1)*	-0.505	0.604	0.257	-2.1	0.049
*Cfa.16217.1.S1_s_at: (CR2)*	-0.527	0.59	0.272	-1.94	0.052
*Cfa.182.1.S2_at: (HLA-DQA1)*	-0.467	0.627	0.241	-1.93	0.053
*Cfa.10937.1.S1_at: (LMO2)*	-0.676	0.509	0.354	-1.91	0.056
*CfaAffx.6511.1.S1_at: (MYCN)*	-1.19	0.304	0.63	-1.89	0.059
*Cfa.15639.1.A1_at: (TP53)*	-0.373	0.688	0.202	-1.85	0.064
*Cfa.5536.1.A1_at: (MYCN)*	-0.781	0.458	0.443	-1.76	0.078
*CfaAffx.18218.1.S1_at: (CR2)*	-0.391	0.677	0.225	-1.74	0.083
*CfaAffx.18149.1.S1_s_at: (CR2)*	-0.429	0.651	0.248	-1.73	0.083
*CfaAffx.18202.1.S1_s_at: (CR2)*	-0.454	0.635	0.272	-1.67	0.095
*Cfa.19191.1.S1_at: (PDE4B)*	-0.857	0.425	0.518	-1.65	0.098
*CfaAffx.4400.1.S1_at: (PAX5)*	-1.65	0.192	1.05	-1.58	0.11
*CfaAffx.11868.1.S1_at: (MYBL1)*	-1.21	0.297	0.773	-1.57	0.12
*Cfa.3707.1.A1_s_at: (FN1)*	0.622	1.86	0.415	1.5	0.13
*Cfa.3800.2.S1_at: (CD44)*	0.226	1.25	0.14	1.61	0.11
*CfaAffx.11235.1.S1_s_at: (CD44)*	0.259	1.3	0.142	1.82	0.068
*Cfa.127.1.S1_s_at: (PLAU)*	0.884	2.42	0.463	1.91	0.057
*Cfa.3707.2.S1_at: (FN1)*	0.277	1.32	0.129	2.15	0.031
*CfaAffx.22155.1.S1_s_at: (FN1)*	0.347	1.41	0.154	2.26	0.024
*Cfa.3707.3.S1_s_at: (FN1)*	0.827	2.29	0.336	2.46	0.014
*GSE39365 dataset*					
*Cfa.15826.1.S1_s_at: (BIRC5)*	-0.832	0.435	0.477	-1.74	0.081
*Cfa.16248.1.S1_at: (CCND1)*	-0.923	0.397	0.54	-1.71	0.088
*Cfa.5536.1.A1_at: (MYCN)*	-2.44	0.0875	1.44	-1.69	0.091
*Cfa.10937.1.S1_at: (LMO2)*	-0.825	0.438	0.516	-1.6	0.11
*Cfa.3619.1.S1_at: (CD38)*	-0.804	0.448	0.512	-1.57	0.12
*Cfa.3619.1.S1_s_at: (CD38)*	-0.605	0.546	0.39	-1.55	0.12
*CfaAffx.11868.1.S1_at: (MYBL1)*	-1.76	0.171	1.16	-1.52	0.12
*Clinical features (GSE39365 dataset)*					
*Age at diagnosis (years)*	0.0774	1.08	0.0893	0.866	0.39
*Sex*	0.165	1.18	0.576	0.287	0.77
*Grade*	-1.04	0.354	0.68	-1.53	0.13

Genes with z score higher than 1.5 or lower than -1.5 were listed. Exp (coef) indicates hazard ratio. Positive and negative z score denotes shorter and longer survival time respectively

**Table 3:** Multivariate Cox proportional-hazard analysis of B-cell lymphoma prognostic gene signatures in GSE43664 and GSE39365 datasets

	Coef	Exp (coef)	SE (coef)	z score	*P*
*GSE43664 dataset*					
*CCND1*	-0.72	0.487	0.353	-2.041	0.041
*GSE39365 dataset*					
*BRICS5*	-2.322	0.098	0.834	-2.785	0.0054
*CCND1*	-3.017	0.0489	1.427	-2.114	0.035

Exp (coef) indicates hazard ratio

**Fig. 1:**
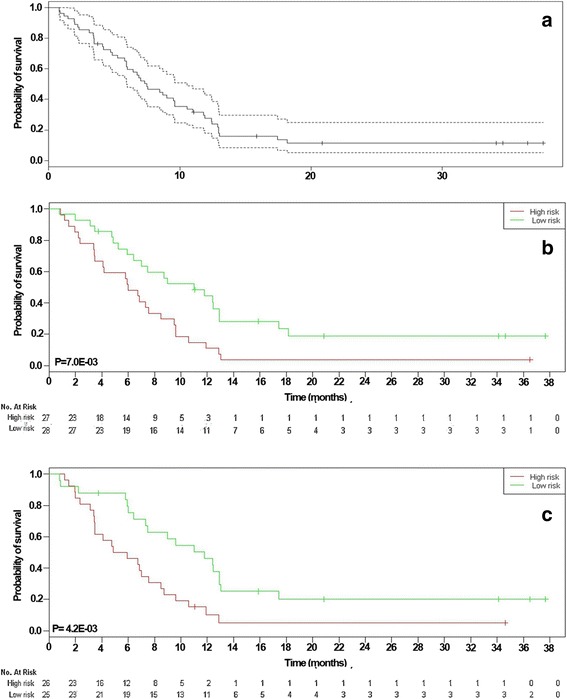
Survival analysis for evaluation of the correlation between GSE39365 prognostic genes and DFS time in GSE43664 dataset. Panel **a** indicated Kaplan-Meier estimate with 95% confidence bound in GSE43664 dataset. There was significant correlation between DFS with *CCND1* (**b**) (*P* = 0.007) and *BIRCS5* (**c**) (*P* = 0.042). *Green* and *red lines* indicated samples had higher and lower expression value than median value respectively

**Fig. 2:**
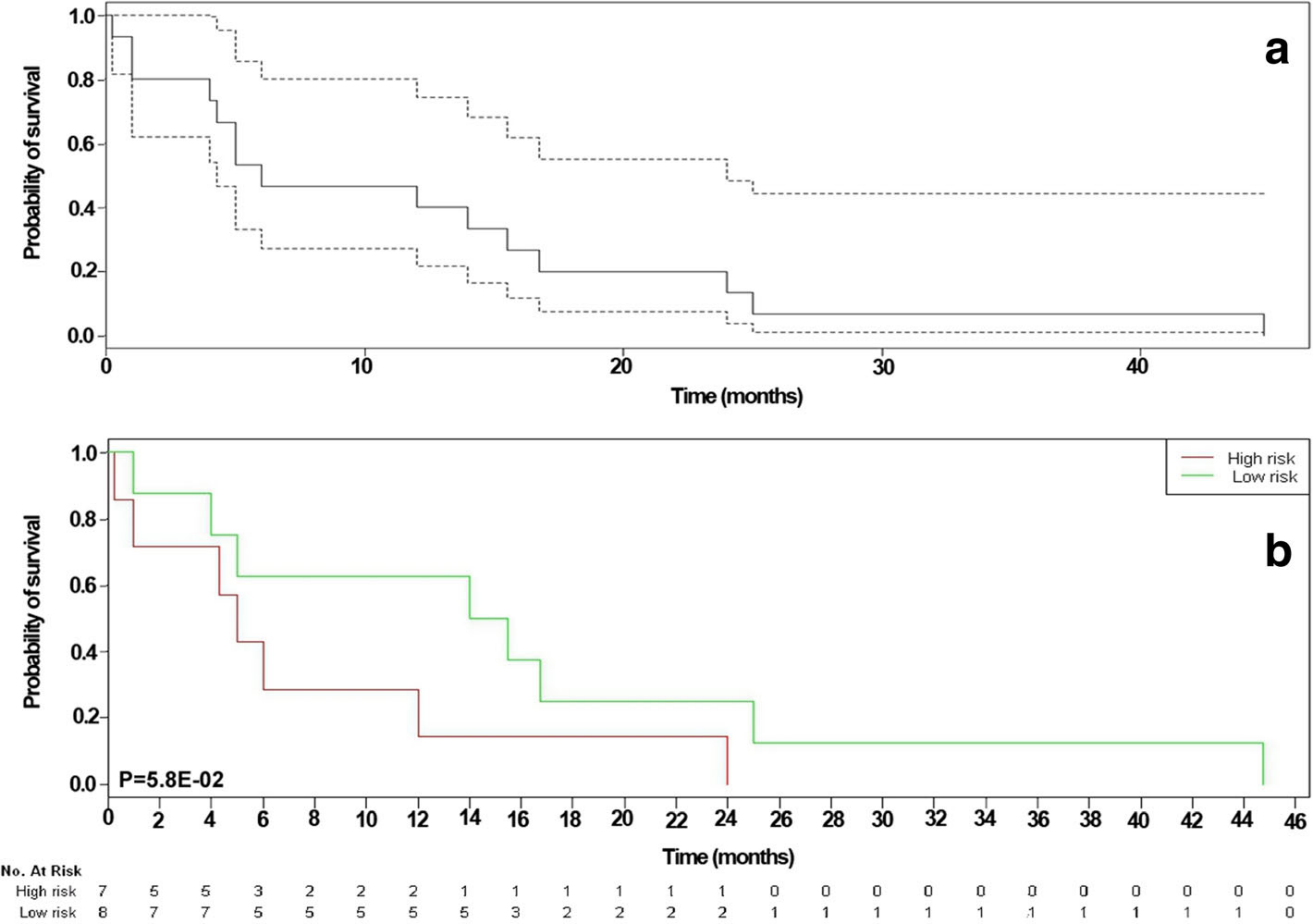
Survival analysis for evaluation of the correlation between GSE43664 prognostic gene and DFS time in GSE39365 dataset. Panel **a** indicated Kaplan-Meier estimate with 95% confidence bound in GSE39365 dataset. There correlation between DFS with *CCND1* (**b**) tended to be significant (*P* = 0.058). *Green* and *red lines* indicated samples had higher and lower expression value than median value respectively

Appropriate external validation was confirmed by validating the prognostic gene(s) in each group in the other group. The correlation between *CCND1* and *BIRCS5* expression and DFS time was tested using the Kaplan-Meier estimator and log-rank test. The patients were divided into high-risk and low-risk groups based on the median of the *CCND1* and *BIRCS5* expression values, and their survival durations were compared using the log-rank test. High-risk and low-risk groups had expression values lower than and higher than the median value respectively. The DFS time in the GSE43664 dataset was statistically different in the survival curves constructed based on *CCND1* (*P* = 0.007) and *BIRCS5* (*P* = 0.0042) expressions (Fig. 1 b and c). However, the DFS time in high-risk and low-risk groups of the GSE39365 dataset tended to be significant (*P* = 0.058) (Fig. 2 b). Additionally, the expression levels of *CCND1* and *BIRCS5* were tested in the GCB-like and ABC-like groups. To that end, the samples were first classified into two groups based on 1,180 canine-specific probe sets. Then, the expression level of *CCND1* was compared between the two groups. For the GSE43664 dataset, a clear clustering pattern was reconstructed (Additional file 1: Figure S1), while the GCB-like and ABC-like groups were not clearly created for the GSE39365 dataset maybe because of its small sample size (Additional file 1: Figure S2). Hence, the T-test analysis was performed only on the GSE43664 dataset and reveled that the differences between *CCND1* expression in the GCB-like (mean ± SD = 8.03 ± 0.86) and ABC-like (mean ± SD = 7.7 ± 0.54) groups tended to be significant (*P* = 0.052) while *BIRCS5* expression in the GCB-like (mean ± SD = 5.32 ± 0.21) and ABC-like (mean ± SD = 5.26 ± 0.23) groups wasn’t significant (*P* = 0.36).

**Fig. 3:**
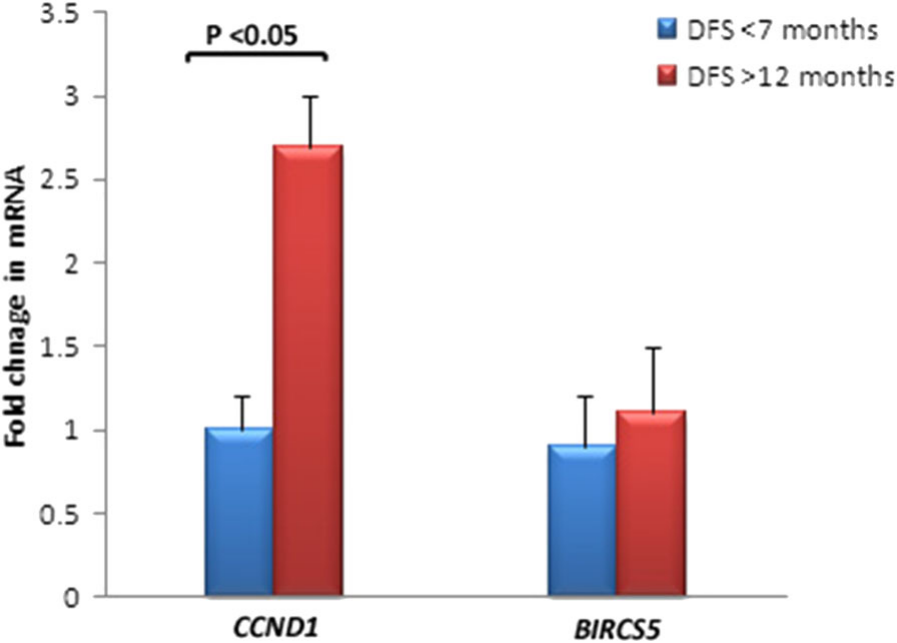
Quantitative real-time PCR (qRT-PCR) analysis of the prognostic genes. Gene expression level of *CCND1* was significantly higher in patients with long DFS time (>12 months) than ones with short DFS time (<7 months). No significant difference was detected in *BIRCS5* expression level between two groups

qRT-PCR analysis confirmed *CCND1* as final prognostic gene because *CCND1* expression was significantly higher in the dogs with DFS >12 months than the dogs with DFS <7 months while expression level of the *BIRCS5* wasn’t significantly different between two groups (Fig. 3).

As was expected in light of our literature review [2, 6], clinical characteristics such as age, sex, and tumor grade were not significant predictor components in canine B-cell lymphoma. More statistical details about the patients' clinical characteristics are summarized in Table 2.

## Discussion

Although prognostic gene genes for human NHL and especially DLBCL have been meticulously investigated by various researchers, there is little information on molecular prognostic genes for canine B-cell lymphoma. For example, Rosenwald et al. (2002) [14] proposed germinal-center B-cell, MHC class II, lymph-node, and cell proliferation signatures as a molecular profiling for predicting progression-free survival after chemotherapy in patients with DLBCL. HLA-DPα, HLA-DQα, HLA-DRα, and HLA-DRβ as members of the MHC class II module; BCL-6 as a member of the germinal-center B-cell module; fibronectin, α-Actinin, connective-tissue growth factor, urokinase plasminogen activator, collagen type IIIα1, and KIAA0233 as members of the lymph-node module; and E21G3, c-myc, and NPM3 as members of the proliferation module constituted the more prominent elements of prognostic signatures [14]. Furthermore, Lossos et al. (2004) [13] proposed a complex of LMO2, FN1, BCL6, SCYA3, CCND2, and BCL2 as a suitable predictor in patients with DLBCL, independent of the International Prognostic Index (IPI). In addition, the authors reported HGAL and BCL6 as predictors of overall survival, independent of the IPI [26, 27]. A comprehensive list of the prognostic genes in human B-cell lymphoma and related studies is presented in the Materials/Methods (Table 1).

The current study utilized human B-cell lymphoma prognostic genes so as to detect valuable genes that can serve as prognostic predictors in canine B-cell lymphoma. There is currently no counterpart for the IPI as regards canine lymphoma inasmuch as the prognostic efficacy of the IPI is evaluated alongside molecular genes. Among genes analyzed in our investigation, *CCND1* was found to be the most appropriate prognostic factor. *CCND1* was confirmed in both datasets while BIRCS5 was solely verified in one dataset. Additionally qRT-PCR verified *CCND1* prognostic efficacy.

*CCND1*, encoding cyclin D1 protein, plays a critical role in the cell cycle machinery; i.e. in G1-S transition. The overexpression of *CCND1* has been indicated in various human B-cell lymphoma subtypes, including mantle cell lymphoma (MCL) [28, 29], DLBCL [30–32], and plasma cell myeloma [33]. Nevertheless, the overexpression of cyclin D1 is regarded as an unusual characteristic in human DLBCL [34, 35]. In general, cyclin D1 has been proposed as the most critical prognostic gene majorly in MCL [36, 37] and seldom for other human B-cell lymphoma subtypes. In one study, cyclin D1 was verified as an independent prognostic factor from the IPI and 5-year overall survival was significantly higher in cyclin D1-negative MCL than cyclin D1-positive MCL (86% vs. 30%) [37]. Furthermore, the m-RNA level of *CCND1* in blood and bone marrow has been proposed as an appropriate prognostic factor in patients with MCL [38]. Cyclin D1 overexpression showed a correlation with longer survival in breast carcinoma [39] and colorectal cancer [40]. The results of our study demonstrate that *CCND1* is a favorable potential prognostic predictor for canine B-cell lymphoma. The results of the present study confirm that *CCND1* is an important potential prognostic gene in canine B-cell lymphoma (especially DLBCL subtype) and should, accordingly, be considered for further investigation in future studies. There is no significant comparable data about prognostic efficacy of the *CCND1* in human DLBCL while studied samples in our study were majorly DLBCL. In an study by lossos et al [13], although univariate Cox proportional-hazard analysis revealed that *CCND1* was a genes with negative z score (longer survival), *CCND1* didn’t reach a significant level for entering final multivariate analysis. Additionally previous investigation revealed that patients with cyclin-D1+ CD5+ DLBCL tended to be associated with inferior survival, but the correlation was not statistically significant [32]. Discrepancy between prognostic efficacy of the *CCND1* in human (especially MCL) and canine B-cell lymphoma can be described in some ways. This discrepancy may stem from the use of different methods for the analysis of *CCND1* expression (e.g. imunohistochemistry, Western blotting, or gene expression analysis) [39, 40] or may related to the species-dependent characteristics. Relationship between *CCND1* expression and survival time in human MCL was evaluated using immunophenotyping methods [37], while we used a gene expression profiling approach in our study. Additionally, some obvious reverse findings have been found between canine B-cell lymphoma and human counterpart. For example, previous investigation indicated that in contrast to human DLBCL, BCL6 and MUM1/IRF4 rarely expressed in canine B-cell lymphoma [16]. Moreover, an inverse expression pattern for p65 and p52 were found in canine and human DLBCL [41]. Furthermore, some potential confounders such as microsatellite instability (MSI), the CpG island methylator phenotype (CIMP), and BRAF mutation have been suggested as another source of the inconsistent findings regarding association between *CCND1* expression and clinical outcome [40]. These genetic aberrations haven’t been examined in canine B-cell lymphoma.

Another gene regarded as a prognostic factor in our study was *BIRCS5* (*survivin*), but it wasn’t confirmed in final qRT-PCR assay. *BIRCS5* is one of the most important inhibitors of apoptosis proteins (IAP) involved in the inhibition of induced cell death in vitro and in vivo [42]. Previous investigations have revealed that overall survival is significantly shorter in patients with high *survivin* expression in patients with MCL [43] and DLBCL [44]. Analysis has confirmed that *survivin* can play a role in the prediction of survival independent of the IPI in DLBCL cases [44, 45]. Be that as it may, some authors have indicated that there is no correlation between *survivin* expression and survival or response to treatment in patients with DLBCL [46]. The overexpression of *survivin* in other cancers such as colorectal cancer and neuroblastoma is associated with higher proliferation activity and higher relapse rate [47, 48]. In our study, although *BIRCS5* was found to be a predictor of DFS in canine B-cell lymphoma in multivariate Cox proportional-hazard analysis, this gene wasn’t considered as suitable prognostic factor because its expression level wasn’t significantly different between patients with long or short survival time.

## Conclusions

To the best of our knowledge, this has been one of the few studies to explore prognostic genes in canine lymphoma using gene expression data analysis. Although microarray data from human cancers are very extensive and informative, microarray data related to animal cancers are rare and incomplete. When mining microarray databases such as GEO and ArrayExpress, there are limited studies exploring canine cancers using gene expression profiling. Similarly, there is same problem with the canine lymphoma. Our mining in the databases provided three datasets (GSE43664, GSE39365, and GSE30881) with ideal sample size on canine B-cell lymphoma, where clinical metadata (including survival time) haven’t been provided for GSE30881 dataset. Therefore, we excluded this dataset and used other two datasets. However, to gain more robust and reliable results, both datasets were used as training and validation groups. Although our results may affect by small sample size of a dataset, we hope that with extending larger canine datasets, future studies by our group or other veterinary oncology researchers will provide more remarkable findings. In conclusion, although the results of the present study reveal *CCND1* as a potential prognostic factor in canine B-cell lymphoma, further studies on more extensive gene expression databases are required to clarify other prognostic genes which can be used as robust survival predictors.

### Ethical approval

All applicable international, national, and/or institutional guidelines for the care and use of animals were followed.

## Additional file

Additional file 1: Additional figures and tables. (DOCX 910 kb)

## Abbreviations

ABC-like: Activated B-cell like
DFS: Disease-free survival
DLBCL: Diffuse large B-cell lymphoma
GCB-like: Germinal center B like
qRT-PCR: Quantitative real-time PCR

## References

[1] MacEwen EG (1990). Spontaneous tumors in dogs and cats: models for the study of cancer biology and treatment. Cancer Metastasis Rev.

[2] Jacobs RM MJ, Valli VE, Meuten DJ (2002). Hemolymphatic system. Tumors in Domestic Animals edn.

[3] McCaw DL, Chan AS, Stegner AL, Mooney B, Bryan JN, Turnquist SE, Henry CJ, Alexander H, Alexander S (2007). Proteomics of Canine Lymphoma Identifies Potential Cancer-Specific Protein Markers. Clin Cancer Res.

[4] Vail DM ME, Young KM, Withrow SJ, Vail DM, Page RL (2001). Canine lymphoma and lymphoid leukemias. Withrow and MacEwen's Small Animal Clinical Oncology edn.

[5] Kiupel M, Teske E, Bostock D (1999). Prognostic factors for treated canine malignant lymphoma. Vet Pathol.

[6] Frantz AM, Sarver AL, Ito D, Phang TL, Karimpour-Fard A, Scott MC, Valli VEO, Lindblad-Toh K, Burgess KE, Husbands BD (2013). Molecular profiling reveals prognostically significant subtypes of canine lymphoma. Vet Pathol.

[7] Teske E, van Heerde P, Rutteman GR, Kurzman ID, Moore PF, MacEwen EG (1994). Prognostic factors for treatment of malignant lymphoma in dogs. J Am Vet Med Assoc.

[8] Löhr CV, Teifke JP, Failing K, Weiss E (1997). Characterization of the proliferation state in canine mammary tumors by the standardized AgNOR method with postfixation and immunohistologic detection of Ki-67 and PCNA. Vet Pathol.

[9] Rmi J, Pg M, Crocker J, Mj L (1990). Sequential demonstration of nucleolar organizer regions and Ki67 immunolabelling in non-Hodgkin's lymphomas. Clinical & Laboratory Haematology.

[10] Yekeler H, Ozercan MR, Yumbul AZ, Ağan M, Ozercan IH (1993). Nucleolar organizer regions in lymphomas: a quantitative study. Pathologica.

[11] Vail DM, Kisseberth WC, Obradovich JE, Moore FM, London CA, MacEwen EG, Ritter MA (1996). Assessment of potential doubling time (Tpot), argyrophilic nucleolar organizer regions (AgNOR), and proliferating cell nuclear antigen (PCNA) as predictors of therapy response in canine non-Hodgkin's lymphoma. Exp Hematol.

[12] Alizadeh AA, Eisen MB, Davis RE, Ma C, Lossos IS, Rosenwald A, Boldrick JC, Sabet H, Tran T, Yu X (2000). Distinct types of diffuse large B-cell lymphoma identified by gene expression profiling. Nature.

[13] Lossos IS, Czerwinski DK, Alizadeh AA, Wechser MA, Tibshirani R, Botstein D, Levy R (2004). Prediction of survival in diffuse large-B-cell lymphoma based on the expression of six genes. N Engl J Med.

[14] Rosenwald A, Wright G, Chan WC, Connors JM, Campo E, Fisher RI, Gascoyne RD, Muller-Hermelink HK, Smeland EB, Giltnane JM (2002). The use of molecular profiling to predict survival after chemotherapy for diffuse large-B-cell lymphoma. N Engl J Med.

[15] Shipp MA, Ross KN, Tamayo P, Weng AP, Kutok JL, Aguiar RCT, Gaasenbeek M, Angelo M, Reich M, Pinkus GS (2002). Diffuse large B-cell lymphoma outcome prediction by gene-expression profiling and supervised machine learning. Nat Med.

[16] Richards KL, Motsinger-Reif AA, Chen H-W, Fedoriw Y, Fan C, Nielsen DM, Small GW, Thomas R, Smith C, Dave SS (2013). Gene profiling of canine B-cell lymphoma reveals germinal center and postgerminal center subtypes with different survival times, modeling human DLBCL. Cancer Res.

[17] Gautier L, Cope L, Bolstad BM, Irizarry RA (2004). affy--analysis of Affymetrix GeneChip data at the probe level. Bioinformatics.

[18] Schroder MS, Culhane AC, Quackenbush J, Haibe-Kains B (2011). survcomp: an R/Bioconductor package for performance assessment and comparison of survival models. Bioinformatics.

[19] Simon R (2005). Roadmap for developing and validating therapeutically relevant genomic classifiers. J Clin Oncol.

[20] Schetter AJ, Nguyen GH, Bowman ED, Mathé EA, Yuen ST, Hawkes JE, Croce CM, Leung SY, Harris CC (2009). Association of inflammation-related and microRNA gene expression with cancer-specific mortality of colon adenocarcinoma. Clin Cancer Res.

[21] Mey U, Hitz F, Lohri A, Pederiva S, Taverna C, Tzankov A, Meier O, Yeow K, Renner C. Diagnosis and treatment of diffuse large B-cell lymphoma. Swiss Med Wkly. 2012;142. 10.4414/smw.2012.13511 22290632

[22] Floratos A, Smith K, Ji Z, Watkinson J, Califano A (2010). geWorkbench: an open source platform for integrative genomics. Bioinformatics.

[23] Livak KJ, Schmittgen TD (2001). Analysis of relative gene expression data using real-time quantitative PCR and the 2(-Delta Delta C(T)) Method. Methods.

[24] Klopfleisch R, Lenze D, Hummel M, Gruber AD. Metastatic canine mammary carcinomas can be identified by a gene expression profile that partly overlaps with human breast cancer profiles. BMC Cancer. 2010;10(1):618. 10.1186/1471-2407-10-618 PMC2994823 21062462

[25] Swerdlow S, Campo E, Harris NL, Jaffe ES, Pileri SA, Stein H, Thiele J, Vardiman JW (2008). WHO Classification of Tumours of Haematopoietic and Lymphoid Tissue, 4 edition edn.

[26] Lossos IS, Alizadeh AA, Rajapaksa R, Tibshirani R, Levy R (2003). HGAL is a novel interleukin-4-inducible gene that strongly predicts survival in diffuse large B-cell lymphoma. Blood.

[27] Lossos IS, Jones CD, Warnke R, Natkunam Y, Kaizer H, Zehnder JL, Tibshirani R, Levy R (2001). Expression of a single gene, BCL-6, strongly predicts survival in patients with diffuse large B-cell lymphoma. Blood.

[28] de Boer CJ, van Krieken JH, Kluin-Nelemans HC, Kluin PM, Schuuring E (1995). Cyclin D1 messenger RNA overexpression as a marker for mantle cell lymphoma. Oncogene.

[29] Gladkikh A, Potashnikova D, Korneva E, Khudoleeva O, Vorobjev I (2010). Cyclin D1 expression in B-cell lymphomas. Exp Hematol.

[30] Vela-Chávez T, Adam P, Kremer M, Bink K, Bacon CM, Menon G, Ferry JA, Fend F, Jaffe ES, Quintanilla-Martínez L (2011). Cyclin D1 positive diffuse large B-cell lymphoma is a post-germinal center-type lymphoma without alterations in the CCND1 gene locus. Leuk Lymphoma.

[31] Hsiao S-C, Cortada IR, Colomo L, Ye H, Liu H, Kuo S-Y, Lin S-H, Chang S-T, Kuo TU, Campo E (2012). SOX11 is useful in differentiating cyclin D1-positive diffuse large B-cell lymphoma from mantle cell lymphoma. Histopathology.

[32] Zhang A, Ohshima K, Sato K, Kanda M, Suzumiya J, Shimazaki K, Kawasaki C, Kikuchi M (1999). Prognostic clinicopathologic factors, including immunologic expression in diffuse large B-cell lymphomas. Pathol Int.

[33] Troussard X, Avet-Loiseau H, Macro M, Mellerin MP, Malet M, Roussel M, Sola B (2000). Cyclin D1 expression in patients with multiple myeloma. Hematol J.

[34] Juskevicius D, Ruiz C, Dirnhofer S, Tzankov A (2014). Clinical, morphologic, phenotypic, and genetic evidence of cyclin D1-positive diffuse large B-cell lymphomas with CYCLIN D1 gene rearrangements. Am J Surg Pathol.

[35] Ok CY, Xu-Monette ZY, Tzankov A, O'Malley DP, Montes-Moreno S, Visco C, Møller MB, Dybkaer K, Orazi A, Zu Y (2014). Prevalence and clinical implications of cyclin D1 expression in diffuse large B-cell lymphoma (DLBCL) treated with immunochemotherapy: a report from the International DLBCL Rituximab-CHOP Consortium Program. Cancer.

[36] Baldin V, Lukas J, Marcote MJ, Pagano M, Draetta G (1993). Cyclin D1 is a nuclear protein required for cell cycle progression in G1. Genes Dev.

[37] Yatabe Y, Suzuki R, Tobinai K, Matsuno Y, Ichinohasama R, Okamoto M, Yamaguchi M, Tamaru J, Uike N, Hashimoto Y (2000). Significance of cyclin D1 overexpression for the diagnosis of mantle cell lymphoma: a clinicopathologic comparison of cyclin D1-positive MCL and cyclin D1-negative MCL-like B-cell lymphoma. Blood.

[38] Siddon AJ, Torres R, Rinder HM, Smith BR, Howe JG, Tormey CA (2012). Normalized CCND1 expression has prognostic value in mantle cell lymphoma. Br J Haematol.

[39] Reis-Filho JS, Savage K, Lambros MBK, James M, Steele D, Jones RL, Dowsett M (2006). Cyclin D1 protein overexpression and CCND1 amplification in breast carcinomas: an immunohistochemical and chromogenic in situ hybridisation analysis. Mod Pathol.

[40] Ogino S, Nosho K, Irahara N, Kure S, Shima K, Baba Y, Toyoda S, Chen L, Giovannucci EL, Meyerhardt JA (2009). A cohort study of cyclin D1 expression and prognosis in 602 colon cancer cases. Clin Cancer Res.

[41] Mudaliar MAV, Haggart RD, Miele G, Sellar G, Tan KAL, Goodlad JR, Milne E, Vail DM, Kurzman I, Crowther D et al. Comparative gene expression profiling identifies common molecular signatures of NF-κB activation in canine and human diffuse large B cell lymphoma (DLBCL). PLoS ONE. 2013;8(9):e72591. 10.1371/journal.pone.0072591 PMC3762807 24023754

[42] Altieri DC (2003). Validating survivin as a cancer therapeutic target. Nat Rev Cancer.

[43] Martinez A, Bellosillo B, Bosch F, Ferrer A, Marcé S, Villamor N, Ott G, Montserrat E, Campo E, Colomer D (2004). Nuclear Survivin Expression in Mantle Cell Lymphoma Is Associated with Cell Proliferation and Survival. Am J Pathol.

[44] Adida C, Haioun C, Gaulard P, Lepage E, Morel P, Briere J, Dombret H, Reyes F, Diebold J, Gisselbrecht C (2000). Prognostic significance of survivin expression in diffuse large B-cell lymphomas. Blood.

[45] Markovic O, Marisavljevic D, Cemerikic-Martinovic V, Martinovic T, Filipovic B, Stanisavljevic D, Zivković R, Hajder J, Stanisavljevic N, Mihaljevic B (2012). Survivin expression in patients with newly diagnosed nodal diffuse large B cell lymphoma (DLBCL). Med Oncol.

[46] Mitrović Z, Ilić I, Aurer I, Kinda SB, Radman I, Dotlić S, Ajduković R, Labar B (2011). Prognostic significance of survivin and caspase-3 immunohistochemical expression in patients with diffuse large B-cell lymphoma treated with rituximab and CHOP. Pathol Oncol Res.

[47] Islam A, Kageyama H, Takada N, Kawamoto T, Takayasu H, Isogai E, Ohira M, Hashizume K, Kobayashi H, Kaneko Y (2000). High expression of Survivin, mapped to 17q25, is significantly associated with poor prognostic factors and promotes cell survival in human neuroblastoma. Oncogene.

[48] Kawasaki H, Altieri DC, Lu CD, Toyoda M, Tenjo T, Tanigawa N (1998). Inhibition of apoptosis by survivin predicts shorter survival rates in colorectal cancer. Cancer Res.

