# Supplementary material for: Prognostic efficacy of the human B-cell lymphoma prognostic genes in predicting disease-free survival (DFS) in the canine counterpart
Source: BMC Vet Res. 2017 Jan 9;13:17. doi: 10.1186/s12917-016-0919-x (PMC5223581; doi:10.1186/s12917-016-0919-x)
Supplement: Additional file 1: — Additional figures and tables. (DOCX 910 kb) [file 12917_2016_919_MOESM1_ESM.docx]

| **Additional table 1**: Clinical characteristics of the studied samples in GSE43664 dataset | | | | |
| --- | --- | --- | --- | --- |
| number | Accession | Source of sample | Pathology diagnosis | DFS time (month) |
| 1 | GSM1067803 | Fine needle aspirate | unknown | 12.93 |
| 2 | GSM1067804 | Lymph node | DLBCL | 3.10 |
| 3 | GSM1067805 | Lymph node | DLBCL | 4.10 |
| 4 | GSM1067806 | Fine needle aspirate | unknown | 1.47 |
| 5 | GSM1067807 | Lymph node | MZL | 10.57 |
| 6 | GSM1067808 | Lymph node | DLBCL | 12.87 |
| 7 | GSM1067809 | Lymph node | DLBCL | 6.87 |
| 8 | GSM1067810 | Lymph node | DLBCL | 9.50 |
| 9 | GSM1067811 | Fine needle aspirate | unknown | 5.93 |
| 10 | GSM1067812 | Lymph node | DLBCL | 1.93 |
| 11 | GSM1067813 | Lymph node | DLBCL | 12.43 |
| 12 | GSM1067814 | Fine needle aspirate | unknown | 12.93 |
| 13 | GSM1067815 | Lymph node | DLBCL | 11.90 |
| 14 | GSM1067816 | Lymph node | DLBCL | 3.40 |
| 15 | GSM1067817 | Fine needle aspirate | unknown | 6.00 |
| 16 | GSM1067818 | Fine needle aspirate | unknown | 3.47 |
| 17 | GSM1067819 | Lymph node | DLBCL | 4.17 |
| 18 | GSM1067820 | Lymph node | DLBCL | 4.73 |
| 19 | GSM1067821 | Lymph node | DLBCL | 4.87 |
| 20 | GSM1067822 | Fine needle aspirate | unknown | 34.60 |
| 21 | GSM1067823 | Lymph node | DLBCL | 2.00 |
| 22 | GSM1067824 | Lymph node | DLBCL |  |
| 23 | GSM1067825 | Lymph node | DLBCL | 1.17 |
| 24 | GSM1067826 | Lymph node | MZL | 8.47 |
| 25 | GSM1067827 | Lymph node | DLBCL | 2.33 |
| 26 | GSM1067828 | Lymph node | DLBCL | 3.43 |
| 27 | GSM1067829 | Lymph node | DLBCL | 7.00 |
| 28 | GSM1067830 | Lymph node | DLBCL | 9.60 |
| 29 | GSM1067831 | Fine needle aspirate | unknown | 7.57 |
| 30 | GSM1067832 | Fine needle aspirate | unknown | 13.03 |
| 31 | GSM1067833 | Fine needle aspirate | unknown | 17.43 |
| 32 | GSM1067834 | Lymph node | DLBCL | 9.60 |
| 33 | GSM1067835 | Fine needle aspirate | unknown | 7.47 |
| 34 | GSM1067836 | Fine needle aspirate | unknown | 11.80 |
| 35 | GSM1067837 | Fine needle aspirate | unknown | 37.67 |
| 36 | GSM1067838 | Lymph node | MZL |  |
| 37 | GSM1067839 | Lymph node | DLBCL | 5.80 |
| 38 | GSM1067840 | Lymph node | DLBCL |  |
| 39 | GSM1067841 | Lymph node | DLBCL | 6.73 |
| 40 | GSM1067842 | Fine needle aspirate | unknown | 8.70 |
| 41 | GSM1067843 | Lymph node | DLBCL | 3.73 |
| 42 | GSM1067844 | Fine needle aspirate | unknown | 11.00 |
| 43 | GSM1067845 | Lymph node | MZL | 3.47 |
| 44 | GSM1067846 | Lymph node | DLBCL | 15.87 |
| 45 | GSM1067847 | Lymph node | MZL | 11.07 |
| 46 | GSM1067848 | Fine needle aspirate | unknown | 0.87 |
| 47 | GSM1067849 | Fine needle aspirate | unknown | 36.50 |
| 48 | GSM1067850 | Lymph node | DLBCL | 5.93 |
| 49 | GSM1067851 | Lymph node | DLBCL | 34.10 |
| 50 | GSM1067852 | Lymph node | DLBCL | 5.27 |
| 51 | GSM1067853 | Fine needle aspirate | unknown | 6.43 |
| 52 | GSM1067854 | Fine needle aspirate | unknown | 9.00 |
| 53 | GSM1067855 | Lymph node | DLBCL | 0.80 |
| 54 | GSM1067856 | Lymph node | DLBCL | 18.17 |
| 55 | GSM1067857 | Lymph node | DLBCL | 12.40 |
| 56 | GSM1067858 | Lymph node | DLBCL | 7.30 |
| 57 | GSM1067859 | Lymph node | MZL | 20.83 |
| 58 | GSM1067860 | Lymph node | MZL | 2.23 |

| **Additional table 2**: Clinical characteristics of the studied samples in GSE39365 dataset | | | | | | | |
| --- | --- | --- | --- | --- | --- | --- | --- |
| number | Accession | Pathology diagnosis | Breed | DFS time(Months) | Age at diagnosis (Years) | Sex | Grade |
| 1 | AMC33_Canine_2 | DLBCL | Rottweiler | 1 | 4.24 | F | High |
| 2 | AMC72_Canine_2 | MZL | Golden Retriever | 0.25 | 7.32 | F | Low |
| 3 | AMC70_Canine_2 | BL | German Shepherd | 44.75 | 5.53 | F | High |
| 4 | AMC71_Canine_2 | DLBCL | Golden Retriever | 6 | 6.63 | M | High |
| 5 | AMC35_Canine_2 | DLBCL | Labrador | 25 | 11.41 | F | High |
| 6 | AMC43_Canine_2 | MZL | Mastiff |  | 4 | F | Low |
| 7 | AMC67_Canine_2 | DLBCL | Rottweiler | 15.5 | 5 | F | High |
| 8 | AMC23_Canine_2 | MZL | Golden Retriever | 5 | 5.03 | M | Low |
| 9 | AMC73_Canine_2 | DLBCL | Golden Retriever | 4 | 10.69 | F | High |
| 10 | AMC26_Canine_2 | DLBCL | Golden Retriever | 14 | 6.13 | M | High |
| 11 | AMC77_Canine_2 | DLBCL | Boxer | 16.75 | 7.67 | F | High |
| 12 | AMC74_Canine_2 | DLBCL | Golden Retriever | 4.3 | 9.74 | M | High |
| 13 | AMC53_Canine_2 | MZL | Golden Retriever | 5 | 8.62 | F | Low |
| 14 | AMC35_1_Canine_2 | DLBCL | Labrador |  | 11 | F | High |
| 15 | AMC40_Canine_2 | DLBCL | Golden Retriever | 1 | 14.11 | F | High |
| 16 | AMC63_Canine_2 | MZL | Golden Retriever | 12 | 5.19 | F | Low |
| 17 | AMC22_Canine_2 | BL | Golden Retriever | 24 | 2 | M | High |
| 18 | AMC41_Canine_2 | DLBCL | Golden Retriever |  | 14 | M | High |

| **Additional table 3. Primer pairs of qRT-PCR assay** | | | | |
| --- | --- | --- | --- | --- |
|  | Forward primer | Reverse primer | amplicon size(bp) | GenBank Accession number |
| ***HPRT*** | TGGCGACCCGCAGCCCCA | CAAGCCGCTCAGTCCTGTCC | 146 | [**NM_001003357.1**](http://www.ncbi.nlm.nih.gov/nucleotide/50979219?report=genbank&log$=nucltop&blast_rank=1&RID=VNFUS6NR01S) |
| ***CCND1*** | ACGAACTGCTGCAAATGGA | GAGGGTGGGTTGGAAATGA | 199 | **NM_001005757.1** |
| ***BIRCS5*** | CCTGGCAGCTCTACCTCAA | CTCAGTGGGACAGTGGATG | 122 | **NM_001003348.1** |


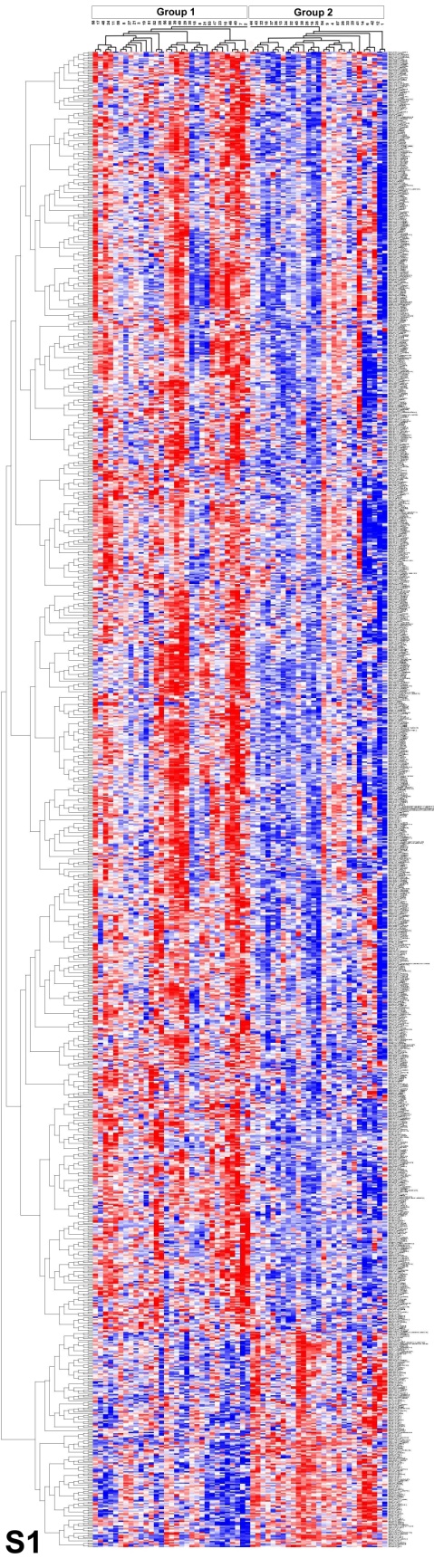


**Additional figures 1:** hierarchical clustering pattern of the studied samples (GSE43664 dataset) using geWorkbench 2.5.1 package. There was a clear clustering pattern for samples in GSE43664 dataset.


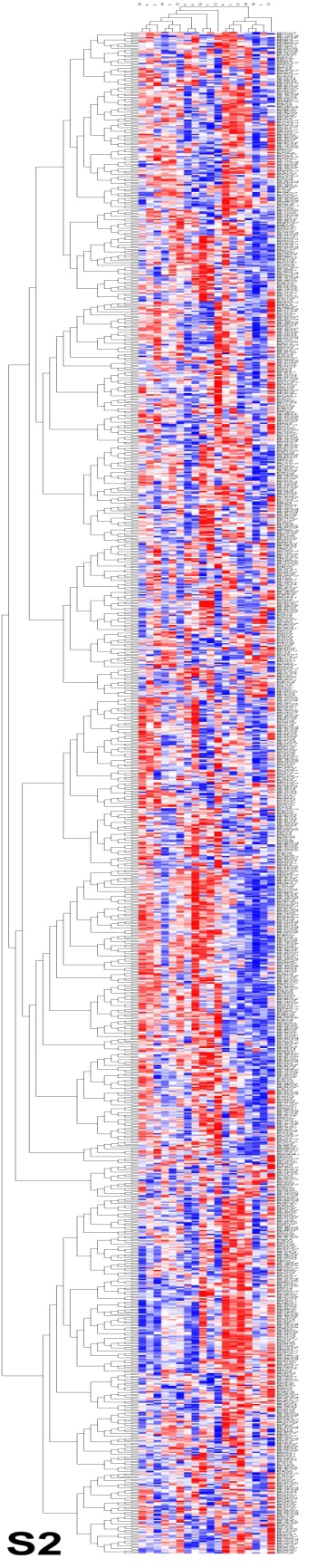


**Additional figures 2:** hierarchical clustering pattern of the studied samples (GSE39365 dataset) using geWorkbench 2.5.1 package. Samples in GSE39365 dataset didn’t indicate a lucid clustering pattern.

**Additional references 1**

1. Gascoyne RD, Adomat SA, Krajewski S, Krajewska M, Horsman DE, Tolcher AW, et al. Prognostic significance of Bcl-2 protein expression and Bcl-2 gene rearrangement in diffuse aggressive non-Hodgkin's lymphoma. Blood 1997; 90:244-51.

2. Kramer MH, Hermans J, Parker J, Krol AD, Kluin-Nelemans JC, Haak HL, et al. Clinical significance of bcl2 and p53 protein expression in diffuse large B-cell lymphoma: a population-based study. J Clin Oncol 1996; 14:2131-38.

3. Hermine O, Haioun C, Lepage E, d'Agay MF, Briere J, Lavignac C, et al. Prognostic significance of bcl-2 protein expression in aggressive non-Hodgkin's lymphoma. Groupe d'Etude des Lymphomes de l'Adulte (GELA). Blood 1996; 87:265-72.

4. Alizadeh AA, Eisen MB, Davis RE, Ma C, Lossos IS, Rosenwald A, et al. Distinct types of diffuse large B-cell lymphoma identified by gene expression profiling. Nature 2000; 403:503-11.

5. Møller MB, Kania PW, Ino Y, Gerdes AM, Nielsen O, Louis DN, et al. Frequent disruption of the RB1 pathway in diffuse large B cell lymphoma: prognostic significance of E2F-1 and p16INK4A. Leukemia 2000; 14:898-904.

6. Rosenwald A, Wright G, Chan WC, Connors JM, Campo E, Fisher RI, et al. The use of molecular profiling to predict survival after chemotherapy for diffuse large-B-cell lymphoma. N Engl J Med 2002; 346:1937-47.

7. Lossos IS, Jones CD, Warnke R, Natkunam Y, Kaizer H, Zehnder JL, et al. Expression of a single gene, BCL-6, strongly predicts survival in patients with diffuse large B-cell lymphoma. Blood 2001; 98:945-51.

8. Lossos IS, Czerwinski DK, Alizadeh AA, Wechser MA, Tibshirani R, Botstein D, et al. Prediction of survival in diffuse large-B-cell lymphoma based on the expression of six genes. N Engl J Med 2004; 350:1828-37.

9. Adida C, Haioun C, Gaulard P, Lepage E, Morel P, Briere J, et al. Prognostic significance of survivin expression in diffuse large B-cell lymphomas. Blood 2000; 96:1921-25.

10. Zhang A, Ohshima K, Sato K, Kanda M, Suzumiya J, Shimazaki K, et al. Prognostic clinicopathologic factors, including immunologic expression in diffuse large B-cell lymphomas. Pathol Int 1999; 49:1043-52.

11. Shaffer AL, Yu X, He Y, Boldrick J, Chan EP, Staudt LM. BCL-6 represses genes that function in lymphocyte differentiation, inflammation, and cell cycle control. Immunity 2000; 13:199-212.

12. Shipp MA, Ross KN, Tamayo P, Weng AP, Kutok JL, Aguiar RCT, et al. Diffuse large B-cell lymphoma outcome prediction by gene-expression profiling and supervised machine learning. Nat Med 2002; 8:68-74.

13. Koduru PR, Raju K, Vadmal V, Menezes G, Shah S, Susin M, et al. Correlation between mutation in P53, p53 expression, cytogenetics, histologic type, and survival in patients with B-cell non-Hodgkin's lymphoma. Blood 1997; 90:4078-91.

14. Drillenburg P, Wielenga VJ. CD44 expression predicts disease outcome in localized large B cell lymphoma. Leukemia : official journal of the Leukemia Society of America, Leukemia Research Fund, UK 1999; 13:1448-55.

15. Krenacs L, Himmelmann AW, Quintanilla-Martinez L, Fest T, Riva A, Wellmann A, et al. Transcription factor B-cell-specific activator protein (BSAP) is differentially expressed in B cells and in subsets of B-cell lymphomas. Blood 1998; 92:1308-16.

16. Thorstenson YR, Shen P, Tusher VG, Wayne TL, Davis RW, Chu G, et al. Global analysis of ATM polymorphism reveals significant functional constraint. Am J Hum Genet 2001; 69:396-412.
